# Orville Horwitz, M. D.

**Published:** 1913-05

**Authors:** 


Orville Horwitz, M. D., Jefferson Medical College, Philadelphia, 1883; emeritus professor of genito-urinary surgery in his alma mater; formerly surgeon to Jefferson Medical College

Hospital, St. Agnes Hospital, the State Hospital for the Insane and Philadelphia General Hospital; consulting surgeon to the Jewish Hospital, formerly a member of the American Medical Association; a member of the Philadelphia College of Physicians, American Surgical Association, American Association of Genito-Urinary Surgeons and American Urological Association, died at his home in Philadelphia, January 28, from spinal sclerosis, aged 52.

